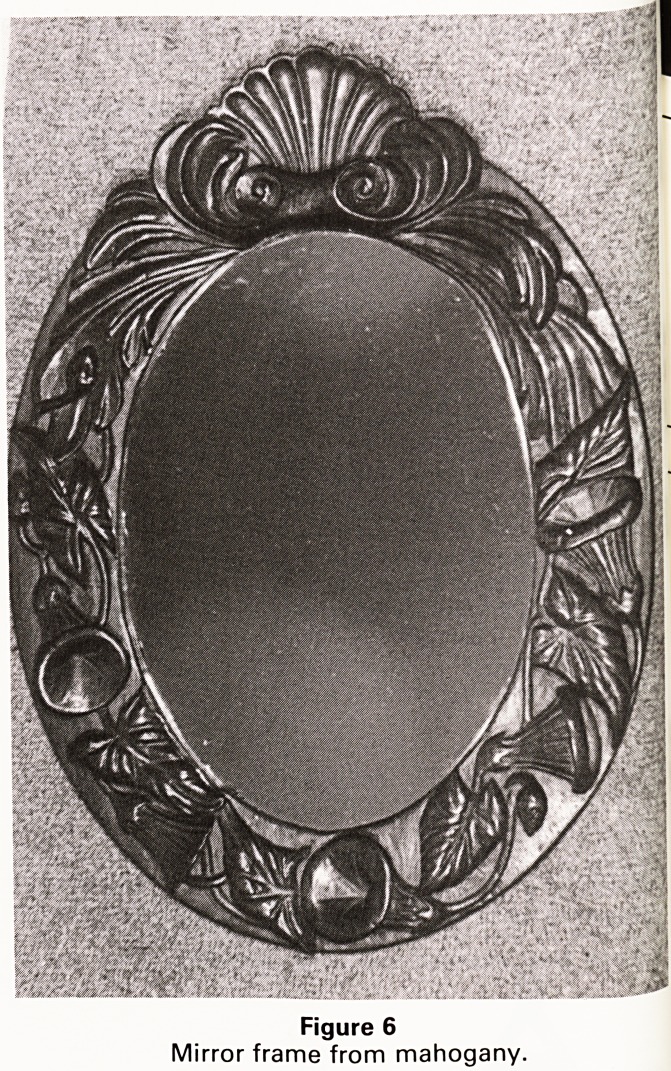# Woodcarving

**Published:** 1987-02

**Authors:** Alec S. Baxter

**Affiliations:** Superintendent Radiographer, Weston-super-Mare Hospitals


### Woodcarving

**Alec S. Baxter**
Superintendent Radiographer, Weston-super-Mare Hospitals

If I were asked when my interest in woodcarving came to life I would tend to say that it was 25 years ago when my then young and indulgent wife presented me with a birthday gift of a dozen woodcarving tools. On reflection, perhaps it was the production of a primitive Spitfire from selected pieces of grandfather's firewood at the age of nine, which first stimulated my enthusiasm. Whatever the start my interest has grown over the years and I continue to take pleasure from the combination of craftmanship and artistic application that woodcarving requires.

My own idea of 'woodcarving' proper is that of the traditional relief style, complete with visible tool marks, which can be found in many old churches. I consider free standing, smooth finished figures, either true to life or abstract, as wood sculpture (see Figure 1). Whatever classification it falls under, the product of the wood craftsman can afford much pleasure both to the producer and (hopefully) the beholder. There is something very satisfying about being able to 'free' a decorative image from within the grain of a block of wood.

A first foray into the world of woodcarving need not be expensive. The tools of the average handyman can be adequate for a beginner, particularly if the project is an abstract form. For these pieces it is the smoothness of line and invitation for tactile appreciation that is required, rather than the intricate changes in the shape and direction of cuts which are demanded by the more traditional styles. A strong bench with the means of firmly securing the wood, leaving both hands free for carving, is one essential for any beginner.

Specialist woodcarving tools are available in a multitude of shapes and sizes, and the most important implements are the gouges. The difference between a woodcarvers gouge and a carpenters gouge is in the position of the bevel of the cutting edge. On a woodcarvers gouge the bevel is on the outside of the curve. This ensures that when being used the tendency is for the gouge to come up out of the wood again rather than bury itself deeper into the block.

There are many types of gouges available with various lengths of blade, shapes of handle, and size and shape of cutting edge. The cutting edge of a gouge may vary not only in width, but in the shape of the curve—from a very shallow almost flat curve (for gentle paring) to a deep 'U' shape (for clearing large blank areas in the design), while the 'V' shaped tools have differing angles for the intricate incisions which provide the fine detail of a design. In other types of gouges the shaft of the blade may be curved backwards or forwards, thus allowing access to the more intricate and partially hidden areas of the carving. Some very specialised gouges have interesting names of Italian influence; one can use fluters or veiners and have Macaroni, Fluteroni and Backeroni gouges on the bench.

Tools which are very specific to the woodcarver and sculptor are the 'rifflers', narrow double ended rasps available in many shapes and sizes. These tools can reach the parts of a carving to which no other tool has access, making them an essential acquisition for the committed carver. Another essential for the enthusiast is a bell shaped mallet or preferably two, one large and another rather smaller.

For the beginner one mallet and as little as half a dozen carefully selected gouges will suffice, extra tools may be purchased as their application is recognised. A full range of tools can be obtained by post or in person from the main specialist supplier Alec Tiranti Limited of Goodge Street, London and Theale in Berkshire.

The would-be carver must also learn to become proficient at sharpening his tools. This skill is best learnt from an experienced fellow carver, but if this is not possible there are a number of useful books available. I learnt my sharpening skills from books together with a great deal of trial and error. When first purchased carving tools do not have the edge sharpened, so that sharpening always precedes carving. In order to get a good working edge on a gouge without destroying the original curve of the tool, a gentle, co-ordinated technique of sliding and rocking is needed. At least two grades of sharpening stone should be used as well as a specially shaped 'slip-stone' to sharpen the internal curves of the gouge. Working with the hardest woods will soon blunt gouges, and the crisp cuts required will be lost; with some projects one can spend longer sharpening the tools than actually using them!

Obviously, the skills of the carver can only be acquired at the bench. The most important skill to master is the use of the mallet, a technique not usually required by the D.I.Y. enthusiast. The mallet must be heavy enough to drive the gouge throught the wood without being too heavy for the constant use. In order to build up a rapport between hand and tool it is useful to practise using gouge and mallet to follow contours on a scrap block of wood.

For the finer shaping of work the hand use of gouges is needed. This is also a two handed technique, the gouge being held firmly and pushed by the right hand with the first and maybe second finger of the left hand resting on the blade to guide the tool on its correct path. As always when handling sharpened tools, the golden rule is to keep both hands, and fingers, behind the cutting edge. It is essential to practise on a block of scrap wood to get the 'feel' of the tools before embarking on any piece of work.

The initial shaping of wood in any project is performed using the largest gouge and mallet; this is known as 'bosting-in'. As the work progresses, smaller, more specific gouges are used to add finer detail and here the hand replaces the mallet.

Obviously, before embarking on a piece of woodcarving, one must acquire some suitable wood. In general the harder timbers are most suitable because they will take a high level of detail and give a good quality finish. The exception to this rule is English Lime; although soft it is probably the best carving wood of all.

When choosing the wood for any project the figuration of the grain must be considered. A large simple lined carving is best done and can be enhanced by using wood with a bold open grain, walnut, oak, mahogany and elm, whereas a portrait could be ruined by selecting wood with such a strong graining. A close grained wood such as lime, apple or box would be best for small fine detailed carvings. In their efforts to acquire suitable wood amateur woodcarvers can reveal themselves to be expert scroungers. A visit to an auction room may lead to the discovery of a heavy and dated piece of furniture of pre-plywood age, which, when stripped of its lifetime of polish, can yield several suitable planks of oak or mahogany. Driftwood is another source of material if one lives near a suitable shoreline. In recent years a drive through the countryside could lead to the acquisition of logs of elm in quite large quantities. When one's hobby is revealed to aquaintances it is pleasantly surprising how many folk house odd pieces of oak or mahogany off-cuts in the back of their garage which are too small for any household use but are ideal for carving.

Alternatively a visit to a timberyard which specialises in hard wood may be necessary. If this is the case it is important to have decided beforehand what size of wood you require; although large quantities of suitable wood will be expensive, off-cuts may be available quite cheaply.

Having gathered together some tools and a piece of wood the amateur carver then faces the trickiest decision of the whole undertaking—choice of subject. Design is at the core of any artistic craft whether the subject is to be taken from life or is to be abstracted. It is impossible to reproduce exactly the texture of a feather or a leaf in wood, even the master of English carving Grinling Gibbons had his limitations. As in all artistic pursuits, it is in the interpretation of the subject and its adaptation to the medium used, that can make the work a success.

The most ubiquitous source of inspiration is nature. Fish and dolphins in particular seem to lend themselves to the efforts of the woodcarver, their smooth surfaces being combined with pleasing shapes and lines.

Unlike the medieval carvers, able only to use subjects from their own everyday experience, present day access to television and books can lend us ideas from a multitude of lands and cultures. Stylised African carving can demonstrate a less complicated approach to what may seem like the incredibly difficult task of creating a figure or a face. For abstract carving one should acknowledge the influences of Henry Moore, and follow his example of collecting inspirational articles from nature, pebbles, bones, and queerly shaped pieces of wood.

Since wood carving is a three-dimensional craft, the design must be made in two planes. For free standing subjects drawings made from four view points will be needed to be transferred onto the block of wood. Detailed drawings and notes need to be kept close to hand, for once work has started on the carving, original markings will be removed with the woodchips. When working on a relief design it must be decided beforehand which areas of the design will remain at which level and to keep a note of this—it is all too easy to casually chip away what was originally destined to be a raised leaf or scroll.

Having given an introduction to woodcarving practices I would like to illustrate my earlier points with example of the finished product.

The large abstract study (Figure 1) is carved from a piece of elm, found in a field whilst helping my daughter, then aged six, to gather dandelions for the family rabbit. The original idea was to produce a simple pierced form, shades of Barbara Hepworth. Having embarked on the project however, the shape of the hole was dictated by the direction of the grain so that the final shape of the piece evolved along lines determined by the wood itself. The smaller abstracts are carved in pine and are exercise pieces; experiments with interfacing surfaces and solid shapes reflecting hollowed shapes. Abstract sculpture allows a greater degree of artistic scope and is generally quicker to achieve than more traditional carving.

The next two carvings illustrate the use of wood to depict quite different textures—feathers and leather (Figure 2).

**Figure 1**
Large abstract (elm) and smaller abstracts (pine).

**Figure 2**
Feathers and leather depicted in wood.

The treecreeper was carved from a piece of oak which was rescued from a sixty year old window sill, and the bird is mounted on a block of yew.

I feel that the grain of oak lends itself to the impression of plumage and is why I chose this material. One of the problems with this type of subject is that of undercutting the wood to a degree which will give the impression that the bird is sitting on the tree trunk without removing so much wood that the carving is not safely supported. Like the woodpecker the treecreeper uses its tail to support itself when feeding, and this offered me an authentic base.

The boot (Figure 2) was a 'fun project' to use up an off-cut from the large abstract; the model was one of my old climbing boots. This subject presented several interesting problems but the final result is a pleasing combination of contour and grain. The boot contains a small jam-jar so that it can be used to hold a flower arrangement. It is a distinct advantage for a carving to have some kind of useful role so that it does not spend its life gathering dust on an obscure shelf or window sill.

The pair of dancing girls (Figure 3) are of walnut purchased at a timber yard. The lifelike posed girl was quite a challenge. Human figures are one of the most difficult undertakings in that they are the one subject that the general public have observed more than anything else, any fault in the proportions and pose of the figure being easily noticed. I realise the faults of this carving, but I rely on other people being either too polite or not observant enough to comment. One problem with figure carving is finding a way of supporting the torso whilst preserving a recognisable human shape. In this figure I utilised the fabric of the long skirt to give continuity from the base for support and stability. The abstract figure was certainly quicker to accomplish, and as a decorative piece I think that it gives more of a feeling of movement, but as a piece of woodcarving accomplished the other figure gave me more satisfaction.

**Figure 3**
Dancing girls.

**Figure 4**
The Coat of Arms of the Society of Radiographers.

Relief carving is my first love, and as I indicated earlier I feel that it is the true craft of woodcarving. My first attempt, over 20 years ago, was the coat of arms of the Society of Radiographers (Figure 4), I aimed to copy the traditional medieval style in fairly shallow relief, using a plank of oak.

The second shield (Figure 5) is the arms of the Chief of the Clan Macmillan inspired by a Scottish holiday and a surname of the clan. I chose sycamore for this piece because it will take much finer detail than oak and gives a smoother finish. A deeper relief gives this shield a much more bold appearance than the first; being a hard close grained wood I spent almost as much time sharpening my tools as using them while working on this project. The background on both these shields has been 'frosted', a multitude of small punch marks that gives texture to the background and hides imperfect levelings.

The oval mirror frame (Figure 6) is mahogany which was donated by a colleague from the back of his garage. The design was original, although the shell shape and acanthus leaves were influenced by the 18th Century style employed by Adam. I had long wanted to try to depict the convolvulus flowers and leaves in a lifelike way. Mahogany was not the easiest wood to use for this kind of subject because the grain has a habit of changing direction; this caused more than a few problems. However, the richness of the colour of this wood does compensate for the problems it caused.

**Figure 5**
The Arms of the Chief of the Clan of Macmillan.

**Figure 6**
Mirror frame from mahogany.

Having completed any carving, it must be finished and polished. Methods chosen depend on personal preference, but the big 'NO' is polyurethene gloss. I have seen several pleasant pieces ruined by a quick coat of varnish. I prefer a simple home made polish of beeswax and turpentine.

To attain the ultra-smooth surfaces which enhance the grain of the wood and the shape of the piece, scraping the wood with a sharp blade or even fine sandpaper or steel wool for some abstract shapes, will give the desired effect. If the wood is then wetted and allowed to dry out the grain will lift slightly, the smoothing process can be repeated to a very worthwhile effect. Several applications of polish will give the depth of sheen which shows the grain and contour of the wood to the best advantage.

Having been persuaded to put pen to paper I have actually been very pleased to share my enjoyment in this craft, and I hope that it will encourage another latent woodcarver to emerge. Most libraries will have books about carving and some adult education centres run evening classes. I have listed below some of the books which I have found most useful and interesting.

History and Practice of Woodcarving, Frederick Oughton, Stobart & Son.

The Craft of Woodcarving, Alan & Gill Bridgewater, David & Charles.

Creative Wood Sculpture, Graeme Bentham, Blandford Press.

## Figures and Tables

**Figure 1 f1:**
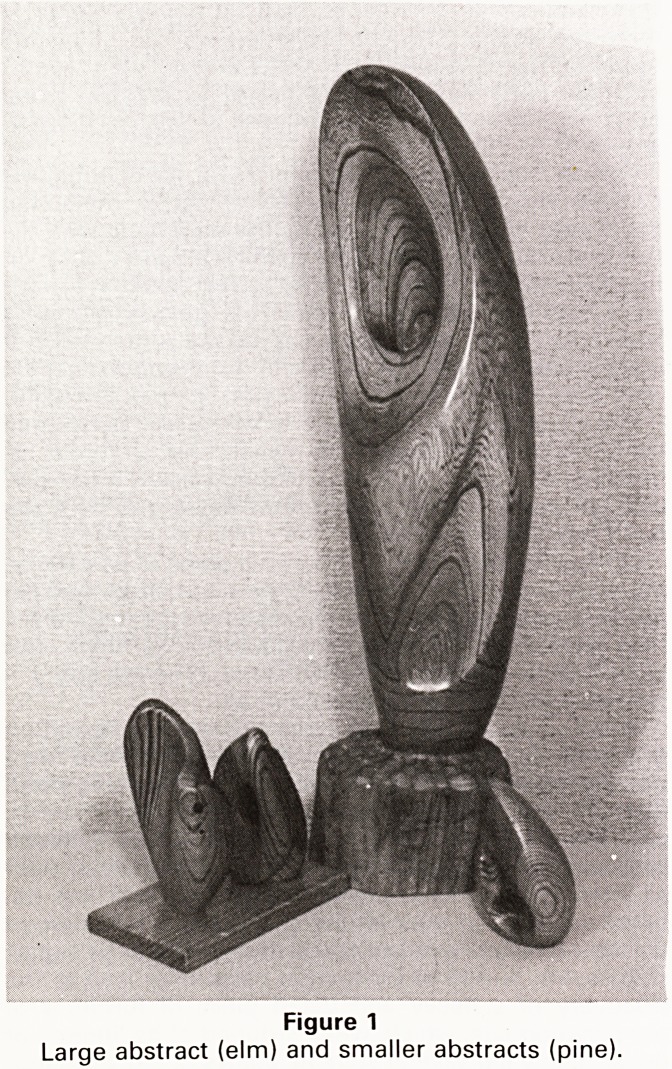


**Figure 2 f2:**
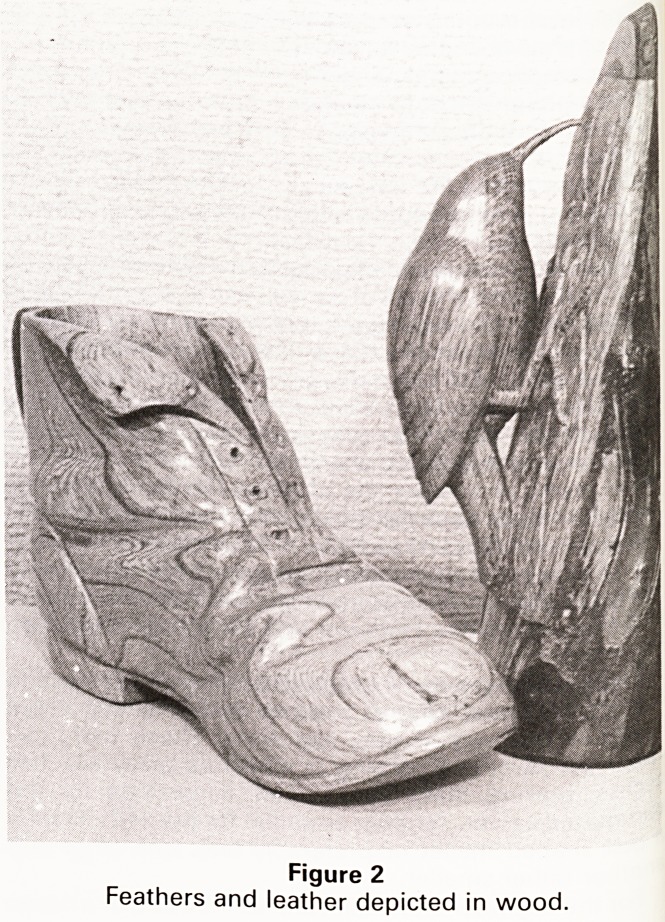


**Figure 3 f3:**
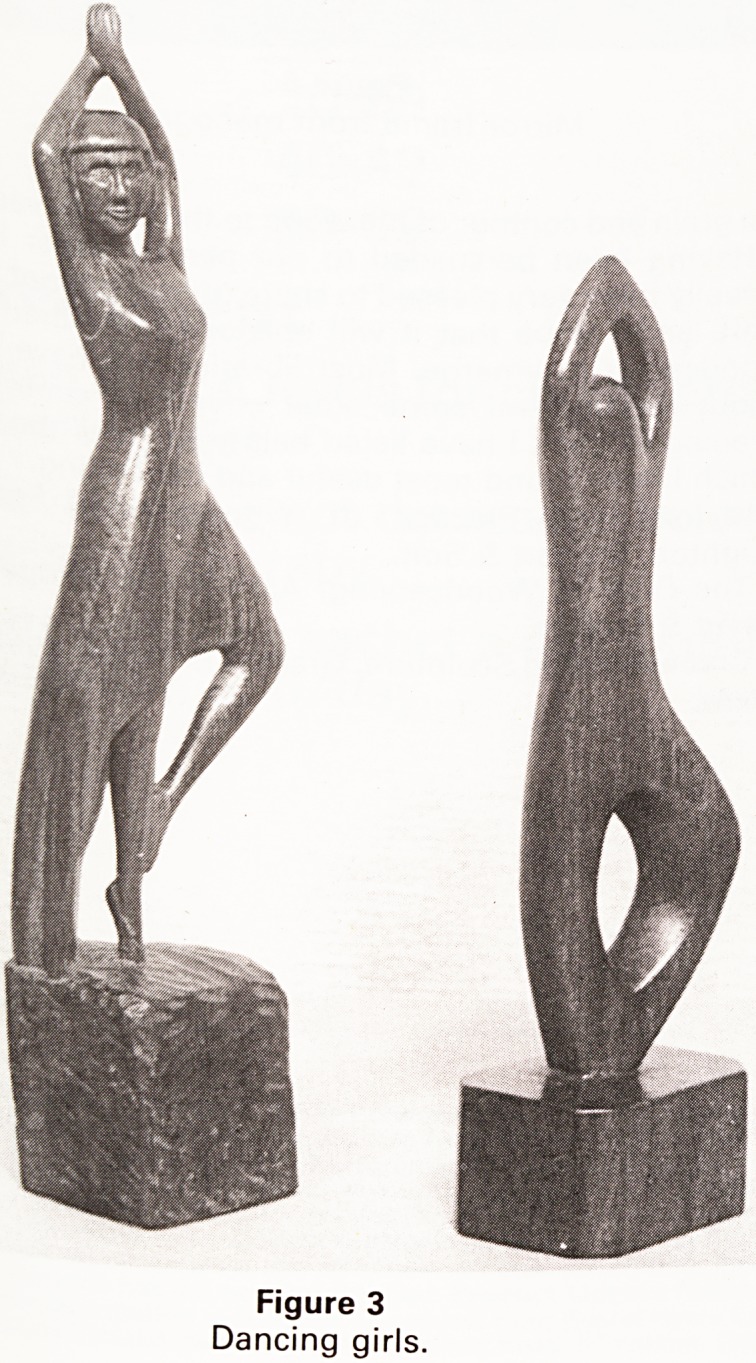


**Figure 4 f4:**
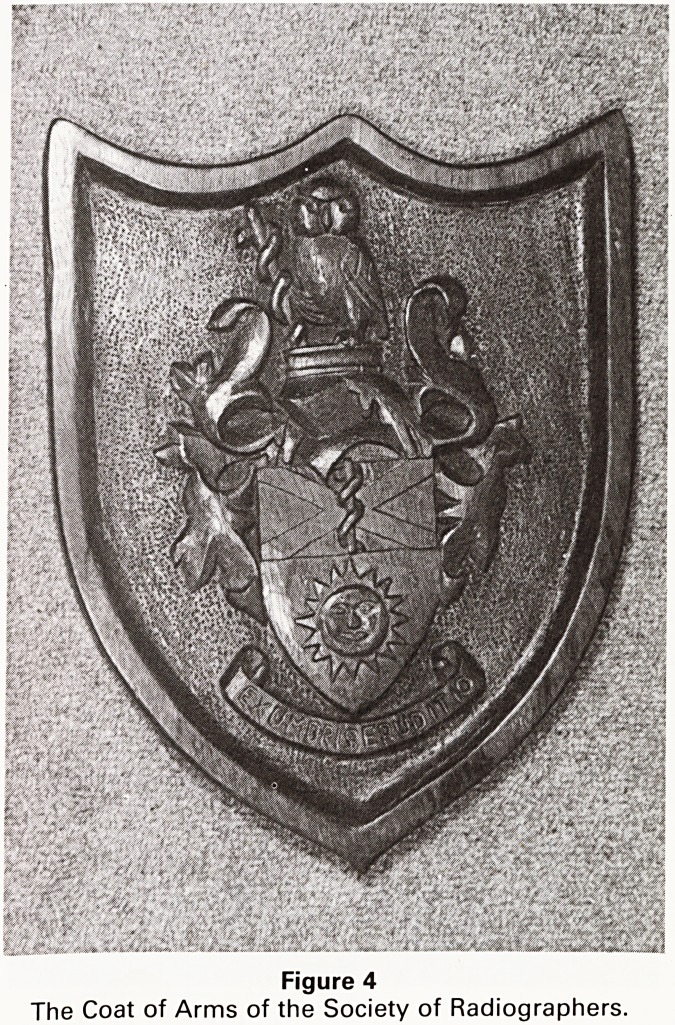


**Figure 5 f5:**
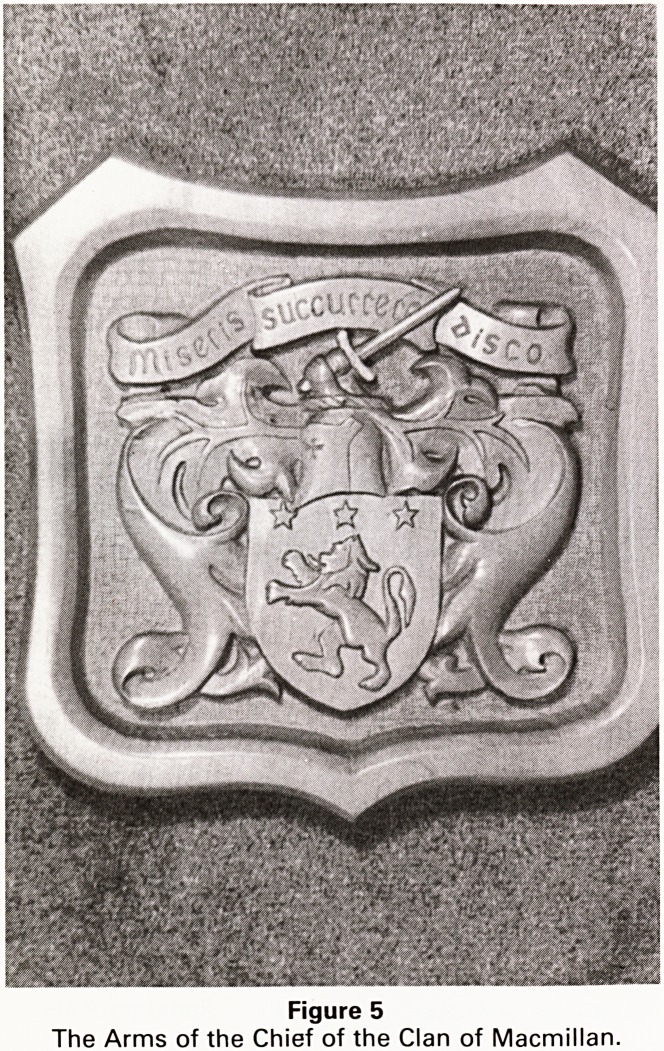


**Figure 6 f6:**